# Kriegel on the Phenomenology of Action

**DOI:** 10.4453/rifp.2016.0026

**Published:** 2016

**Authors:** Joshua Shepherd

**Affiliations:** (α) Jesus College, Oxford Center for Neuroethics, University of Oxford, Turl Street, Oxford OX13DV (UK)

**Keywords:** Agentive Phenomenology, Deciding, Trying, Imperatives

## Abstract

I focus on Uriah Kriegel’s account of conative phenomenology. I agree with Kriegel’s argument that some conative phenomenology is primitive in that some conative phenomenal properties cannot be reduced to another kind of property (e.g., perceptual or cognitive). I disagree, however, with Kriegel’s specific characterization of the properties in question. Kriegel argues that the experience of deciding-and-then-trying is the core of conative phenomenology. I argue, however, that the experiences of trying and acting better occupy this place. Further, I suggest that the attitudinal component of the experiences of trying and acting is not, as Kriegel suggests, best characterized in terms of commitment to the rightness or goodness of the objects of experience. Rather, I argue that the attitudinal component is best characterized in imperatival terms.

## Introduction: Kriegel’s project

In a number of recent papers, and most centrally in his book *The Varieties of Consciousness*,^1^ Uriah Kriegel attempts to map out the nature and structure of conscious experience. In my opinion, his is one of the most exciting projects in the philosophy of mind. Its importance – which I associate with the interest attached to the questions Kriegel is asking, and the insights he is developing in response – is bolstered by Kriegel’s ability to move seamlessly between important historical sources located in the so-called continental traditions of (primarily) Continental Europe, and important contemporary sources located in the so-called analytic tradition of (primarily) the English-speaking nations.

The project involves an attempt to chart a kind of map of the phenomenal realm, guided by a concern to understand the identity and nature of the types of what Kriegel calls primitive phenomenology. For Kriegel, a type of phenomenology is primitive if it is best placed within the second layer of a broader structure of determinable-determinate relations. He is looking for *second*-layer determinables because the first layer is characterized very abstractly:

The highest phenomenal determinable is phenomenality per se (what-it-is-like-ness as such, if you will). It is the phenomenal property that is not a determinate of any other phenomenal property.^2^

Ultimately, at the second layer Kriegel finds at least six primitive types of phenomenology. Perceptual and algedonic phenomenology are accepted as primitive more or less without argument. Argument is given for the existence of cognitive, entertaining, conative, and imaginative phenomenology. Kriegel admits there may be others, and his willingness to consider a range of potential candidates at places in the book lends his discussion considerable interest. The whole thing is worth reading. In what follows, however, I restrict my attention primarily to Kriegel’s arguments on behalf of conative phenomenology – that is, the phenomenology associated with motivation and action.

Kriegel presents his account of conative phenomenology in chapter 2 of *The Varieties of Consciousness*. Two claims are critical, and form the core of the account. First, Kriegel argues that some conative phenomenology is primitive in that some conative phenomenal properties cannot be reduced to another kind of property (e.g., perceptual or cognitive). Second, Kriegel argues for a specific characterization of the properties in question:

the fundamental form of our conative experience is a proprietary phenomenology of deciding-and-then-trying.^3^

In what follows, I first elucidate Kriegel’s arguments for both claims. Next, I assess the arguments. I agree with Kriegel’s irreducibility claim. But I question his characterization of the core properties of conative phenomenology. This disagreement may run deep. For the reasons I offer suggest a separate way to draw the boundaries around conative phenomenology. On this way, the phenomenology of trying and acting may be distinct from conative phenomenology more broadly, with ramifications for how we think of the nature and scope of the primitive kinds of phenomenology Kriegel identifies.

## Kriegel’s conative phenomenology

For Kriegel, conative phenomenology involves phenomenal properties associated with motivation and action. These are properties attached to states and processes described as desiring to A, wishing that P, valuing or disvaluing X, preferring X to Y, intending to A, planning to A, deciding to A, trying to A, doing A, and so on. Kriegel endorses primitivism about conative phenomenology – the claim that «*some* phenomenal property is (i) instantiated by some unquestionably conative state, (ii) not instantiated by any nonconative states, and (iii) irreducible to any (combination of) other phenomenal properties».^4^

Kriegel argues primarily by elimination: he considers and rejects a number of (more or less) plausible proposals for eliminating or reducing conative phenomenology. I am convinced: for purposes of exposition, I consider the last two proposals, which are in my view the most plausible.

The first proposal I will consider has it that the phenomenology of doing something with one’s body – e.g., clenching one’s fist – can be reduced to three things:

(i) tactile phenomenology of one’s hand’s various parts touching each other, (ii) visual (or for that matter cognitive) phenomenology of seeing (or judging) that one caused the fist to clench, and (iii) proprioceptive phenomenology of feeling one’s fist muscles contracting.^5^

Kriegel’s primary problem with this proposal is that it gets the timing of the phenomenology wrong. We do not experience the phenomenology of doing something after it is done, as this account entails. Rather, «the phenomenology of doing the contracting of one’s muscles takes place during, or rather leading up to, the muscle contraction».^6^

The last proposal Kriegel considers is due to William James. The Jamesian proposal appeals not to tactile or proprioceptive feedback, but to anticipative tactile or proprioceptive imagery. As Kriegel notes, «on this view, the key element for capturing the conative dimension of the experience of clenching one’s fist is the feel of imaginatively anticipating one’s fist muscles contracting».^7^

Against this proposal, Kriegel makes two points. First, it again gets the timing of the phenomenology wrong, placing it before the doing takes place. Second, it «seems false to our experience». Kriegel elaborates:

We experience a representation of the act to follow, but also of the act following, and following because we *make* it follow. That is, we experience not only an anticipation of the act, but also the causing of the act in real time.^8^

I agree with Kriegel against James that there is an irreducibly agentive or actional element to the phenomenology. Elsewhere, I have argued that there is no good empirical reason to identify this aspect of phenomenology with anticipative imagery, and furthermore that there is some reason to think that this aspect is at least partially constituted by executive states such as intentions and command signals.^9^ The difficult part is getting the description of this aspect of the phenomenology right. For without a compelling account of the nature of the phenomenology at issue, skeptics will likely find space to dissent.

I turn, then, to Kriegel’s characterization of the core properties of conative phenomenology. The first aspect of this characterization has to do with the nature of conative attitudes.

What seems to characterize conative states is their *value*-*commitment*. To want ice cream, to wish for ice cream, to like ice cream, to approve of ice cream – all these commit to the goodness of ice cream. The notion of goodness at play here is maximally neutral – a kind of completely generic goodness. It covers both moral and other kinds of goodness (e.g., aesthetic). It covers relative goodness (“good for”) and absolute goodness (good *tout court*). It covers the goodness of states of affairs, but also the goodness of actions (“rightness”), mental states (“fittingness”), and persons (“virtue”). It covers intrinsic and final goodness, as well as instrumental goodness. We may call this *generic goodness*, or goodness-G for short. Positive conative states (such as liking or approving of something) are characterized by their goodness-G-commitment; negative ones (disliking, disapproving) by their badness-G-commitment.^10^

Kriegel distinguishes the way that conative attitudes commit to goodness from two other ways of committing to goodness. The first is a belief’s representing-as-true p’s being good. The second is a sensory state’s (e.g., a pain’s) sensuous representing-as-good p. Conative attitudes are nonsensuous, and thus they represent-as-good in a nonsensuous way. Kriegel comments:

If nonsensuous representing-as-good-G is the mark of the conative, then all conscious conative states exhibit what we may call nonsensuous presenting-as-good-G.^11^

The second aspect of Kriegel’s characterization involves the identification of the most fundamental conative states and processes. Here Kriegel draws on the work of Paul Ricoeur, arguing that the core of conative phenomenology is that of deciding-and-then-trying.

Kriegel first considers the experience of deciding, as explicated by Ricoeur.^12^ The phenomenology of deciding is marked by several features. First, decisions are directed to projects represented as in the future. They thereby have a «character of futurity»; second, deciding «presents the project as *in my power*»; third, deciding involves a felt pull to action: «unless a mental state involves a pull to action, it is not a decision».^13^ As Ricoeur has it, in deciding «I feel myself somehow charged, in the way a battery is charged: I have the power to act».^14^ Further, this pull to action is categorical, distinguishing deciding from related states such as desire.

Kriegel claims that the categorical nature of deciding is an attitudinal feature – decisions are characterized by an attitude of commitment to the project or plan that is the decision’s content. How does the specific nature of deciding relate to the general mark of the conative – to what Kriegel calls presenting-as-good-G? Kriegel claims that «decision’s categorical pull-to-action feel casts decision as directed at *the right*».^15^ This is because, for Kriegel, rightness is an attribute of actions, and goodness of states of affairs. And decisions are always about actions. So decisions present-as-right the actions they are about.

According to Kriegel, however, the experience of deciding is incomplete on its own. It requires a complement.

Deciding feels *impatient*: its pull to action is unnerving, strongly calling me to act it out. Not only does the decision dispose me to act, but until the decision is acted upon – until the disposition is manifested – there is a subtly unpleasant feeling of tension in my consciousness. Thus, by its very nature, a decision desperately wants to be realized – realized in action. Phenomenologically, the exercise of the will is not exhausted when a decision has been formed – only when the process of realizing the decision is underway.^16^

Although Kriegel writes in the above passage that a decision wants to be realized in action, he argues that the essential complement of the experience of deciding is an experience of trying. Kriegel is aware that this may seem unnatural. Why think of trying rather than acting as decision’s complement?

Kriegel’s reasoning on this point is as follows. We are attempting to characterize some aspect of phenomenology, and phenomenology is «an entirely mental phenomenon».^17^ Action, however, is not entirely mental. At least in the case of bodily actions, it is constituted in part by bodily movements. Bodily movements on their own lack intentionality, as they fail Chisholm’s test for intentionality. That is, action verbs that involve the body support existential generalization and do not evince substitution failure.

From “Anatole moved his hand” […] one can validly infer “there is something that Anatole moved”; so existential generalization is supported rather than failed. Further, from “Anatole moved his hand” […] in conjunction with “Simone’s favorite object is Anatole’s hand” […] one can validly infer “Anatole moved Simone’s favorite object”; so there is no substitution failure either.^18^

Kriegel favors trying over action as the complement of deciding because trying is, Kriegel avers, entirely mental. Trying passes Chisholm’s test for intentionality. As such, trying is «the mental “core” of action».^19^

As for the phenomenology of trying, Kriegel makes three observations. First, like deciding, trying aims at the right: trying represents-as-right the object of the trying. Second, the experience of trying essentially involves an experience of effort: «trying involves the experience of mobilizing force in the face of resistance».^20^ Third, the experience of trying in some sense satisfies the tension inherent in the experience of deciding. This allows Kriegel to bring together deciding and trying as the joint core of conative phenomenology, as follows:

[T]he feel of deciding to ϕ inherently requires a complement in trying. This marks a deep difference between decision and desire. Since desire’s pull-to-action feel is merely hypothetical, there is nothing phenomenologically problematic about desiring something but trying to do nothing about it. Things are different with decision: given decision’s *categorical* pull-to-action feel, it is strictly impossible that one should decide to ϕ without trying to ϕ. In that respect, the experiences of deciding and trying are, *au fond*, two components of a single experience, which for want of a better term I will call the “phenomenology of deciding-cum-trying”.^21^

So concludes my brief elucidation of Kriegel’s account of the core of conative phenomenology.^22^ There is much to like about this account, and many features of it that I am happy to accept. In what follows, however, I focus on areas of disagreement. A different view of the terrain Kriegel has covered is available. My aim is to shed some light on it so that we may compare alternatives and, if things go well for us, to sharpen our understanding of the terrain.

## Assessing the characterization of conative phenomenology’s core

Recall that Kriegel’s account has two aspects. The first has to do with the nature of conative attitudes. The second has to do with a characterization of the core conative states and processes. I discuss these aspects in reverse order, beginning with the phenomenology of deciding.

I agree with some features of Kriegel’s Ricoeurian account. Conscious deciding has a character of futurity. Conscious deciding involves a felt pull-to-action. (Although I might emphasize that there is an active aspect to this “felt pull”, perhaps better described as a felt charge to act.) Conscious deciding is categorical in nature. But I am not convinced that conscious deciding requires a complement.

Here is why I am not convinced. Action theorists distinguish between distal intentions and proximal intentions. These are intentions to A at some point in the future, and intentions to A now, respectively. Accordingly, we can distinguish distal from proximal decisions. Distal decisions are intentional mental actions of distal intention formation; proximal intentions are intentional mental actions of proximal intention formation. If any conscious decisions require a complement, it is conscious proximal decisions. But – here is a crucial claim in my reasoning – the phenomenology of distal and proximal deciding is the same qua deciding (that is, as regards intention formation). Since distal decisions do not require a complement, then we cannot draw the claim about complement requirement from the phenomenology of deciding.

In my view, the core of the phenomenology of deciding can be described as that of performing the mental action of assenting or committing to a plan.^23^ How best to understand the causal processes that undergird the phenomenology is a difficult issue.^24^ But as for the phenomenology, it seems to me that the mental action of assenting or committing is not irreducible. It involves trying – the mobilizing of effort as Kriegel puts it – and it typically is successful. So the phenomenology of deciding is just the phenomenology of trying to do or of doing a certain thing, namely, deciding.

If this is right, then the core of the phenomenology in question will be reduced to trying, or acting. Certainly Kriegel will opt for trying, for reasons we have seen. But I am not convinced we should dispatch with the phenomenology of acting. As Kriegel recognizes, the view that trying is the mental core of action is open to the following objection. Normally, we do not experience ourselves as trying, but as acting. Indeed, Kriegel attributes to Ricoeur the very plausible observation that

in our actual experience it is action that manifests itself to us first and foremost, while trying is relatively obscured and requires careful and somewhat tutored attention.^25^

In response, Kriegel makes two points. The second is an argument that trying, not acting, is the natural complement of deciding. Since I have argued that deciding needs no complement, and is reducible anyway to trying or acting, I leave this point aside.

The point on which I focus involves an analogy with perception. Kriegel notes that we experience ourselves as seeing the world, even though nothing in the experience guarantees success: «our experience is *in fact* a state which might be either a seeing or a hallucinating».^26^ Similarly, Kriegel notes, for experiences of trying and acting.

When it is successful, our experience of ourselves as acting is veridical, and when it is unsuccessful, nonveridical. It remains that nothing in the conative experience itself guarantees its success, just as nothing in a visual experience guarantees its veridicality. So the experience itself is just a trying.^27^

I fail to see how the lack of a guarantee of success renders the experience itself just a trying. Perhaps Kriegel is thinking that in the absence of a guarantee, trying is all we can *know* that we have done. But again, I fail to see how a lack of knowledge about the experience’s veridicality makes the experience a trying.

Here is one way to think about the mental core of action. Either the common experience attached with acting is an experience of trying or one of acting. Consider Ricoeur’s example of the clenching of a fist. When I consciously and successfully clench the fist, there are different aspects to my phenomenology. I experience directing activity (or mobilizing effort) towards the clenching, and I experience certain things attached to the fist actually clenching. I think it is an open question whether what I experience is best described as a decomposable sum of the experience of trying along with perceptual elements related to the fist actually clenching, or instead best described as a non-decomposable unity of the experience of acting – of my clenching the fist. On either description, the total experience involves perceptual elements.

On the former description, we can phenomenologically separate the trying from the clenching. Thus, even in veridical cases, it seems appropriate to describe the agentive core (if not the mental core) of the action as an experience of trying.^28^ On the latter, it seems inappropriate to do so – the trying and the clenching are unified. I do not consciously try to clench the fist. I consciously clench it. On this description, then, the experience of acting has as much claim to the title “mental core of action” as does the experience of trying. We might, then, have to make room for both.

I turn to a different aspect of Kriegel’s account of conative phenomenology – his characterization of the conative attitudes. I accept that the phenomenology attached with desiring, wishing, hoping, valuing, and preferring commits to the goodness of its objects in the way Kriegel describes. But I am not convinced that the phenomenology attached with trying and acting does so – at least not essentially. Rather than positing conative attitudes as essential to these kinds of experiences, we might posit something akin to imperatival attitudes. Imperatives issue commands.

Drawing on Kriegel’s way of explicating attitudes, imperatival attitudes do not represent-as-good (or represent-as-right) their objects. Instead, they represent-as-to-be-done their objects. The difference here is, in part, that imperatival attitudes contain no value commitment. They are concerned only to command (clusters of) action(s).

I think this imperatival proposal can capture the phenomenology of intending (and thereby a part of the phenomenology of deciding, namely, the part associated with the pull-to-action and the charge-to-act mentioned above). Recall Kriegel’s claim:

If nonsensuous representing-as-good-G is the mark of the conative, then all conscious conative states exhibit what we may call nonsensuous presenting-as-good-G.^29^

*Conscious* conative states nonsensuously *present*-as-good their objects. As I have said, I do not disagree that this is accurate as applied to desiring, preferring, and so on. But in the case of intending, I think we confront a subtly distinct type of phenomenology, characterized by a nonsensuous imperatival attitude.^30^ Conscious intentions nonsensuously present-as-to-be-done their objects.

Something else is needed, however, if we wish to capture trying and acting. This is because in trying and acting, an agent does not experience her attempts or actions as to-be-done – she experiences them as what she is doing. In consciously acting, the agent experiences herself at once fulfilling the command she herself generates and maintains.^31^

It is better to say, then, that the attitude that characterizes the phenomenology of trying and acting is a proprietarily executional attitude. When consciously trying or acting, the agent experiences herself as executing the plan that is her goal: she has an experience of *directing activity* towards goal-fulfillment. This kind of experience makes no comment on the goodness or rightness of the thing done – the experience is only concerned with the doing and with what is being done.

Note how little accepting this proposal would change what Kriegel says about the phenomenology at issue. I have not challenged anything Kriegel says about the phenomenology of deciding; I have argued only that it can be explained in terms of trying or acting. Nor have I challenged much of what Kriegel says about trying. Trying is closely connected to the mobilization of effort, as Kriegel notes. I would resist, however, Kriegel’s claim that «given decision’s *categorical* pull-to-action feel, it is strictly impossible that one should decide to ϕ without trying to ϕ». As applied to proximal decisions, I think it is very rare for an agent to decide to ϕ now without trying to ϕ. But I view these as logically distinct experience-types, and thus as separable in principle. It is not inconceivable, in my view, that an agent could have the experience of deciding to ϕ and then, before a trying to ϕ or a ϕ-ing can begin, change her mind. Sometimes evidence for or against a decision continues to accumulate (via sub-personal assessment mechanisms) after a decision is made, leading to rapid changes of mind.^32^

Even so, my proposal regarding the nature of the attitudes at issue in intending, trying and acting does suggest a disagreement with Kriegel regarding the structure of what Kriegel calls second-layer phenomenal primitives – that is, the kinds of phenomenology that share what-it-is-like-ness and nothing else. If my proposal is right, then what Kriegel identifies as the core of conative phenomenology might be better thought of as the core of a different kind of primitive phenomenology – agentive phenomenology.

This is not to say that conative (or motivational) phenomenology is non-existent. A primitive conative phenomenology might still be associated with experiences that are, as Kriegel notes, fundamentally committed to the value of their objects. Experiences associated with desires, wishes, hopes, and preferences seem to be paradigm examples.

But we might go further than this. In his chapter on emotional phenomenology Kriegel discusses a proposal due to Brentano that lumps together conative and emotional phenomenology insofar as «both frame their object as good».^33^ Kriegel notes that if we accept this proposal, «It would then be natural to hold that experiences exhibiting presenting-as-good form a second-layer phenomenal category on a par with experiences exhibiting presenting-as-true and experiences exhibiting mere-presenting».^34^ Emotional and conative phenomenology would turn out to be different classes of the same second-layer phenomenology – what we might call evaluative phenomenology.

One might go even further than this, arguing that algedonic phenomenology – the phenomenology attached with pleasure and pain – constitutes a third kind of evaluative phenomenology. Certainly it is not implausible to think that the valenced aspects associated with pain and pleasure can be explained via attitudes that sensuously present-as-valenced their objects. Against the backdrop of Kriegel’s bigger project, the possibility seems worth considering.

I cannot defend these possibilities at length here, but it is worth noting that on this proposal, we have an explanation for the sense in which emotional, conative and algedonic states motivate action and feature in deliberation. In virtue of the fact that these experiences frame their objects as good or bad in various ways, these states are capable of informing value-based action and decision. When it comes to the proprietarily agentive experiences of trying and acting, however, something different is going on. The experiences of trying and acting do not directly commit to the goodness or rightness of their objects. Rather, these experiences are concerned only with execution. That our tryings and actings can be rationalized by evaluative states and experiences requires an additional layer of mentality – one not found in the tryings and actings themselves.
